# The pattern of anthrax at the wildlife-livestock-human interface in Zimbabwe

**DOI:** 10.1371/journal.pntd.0008800

**Published:** 2020-10-19

**Authors:** Norman L. Mukarati, Gift Matope, Michel de Garine-Wichatitsky, Daud N. Ndhlovu, Alexandre Caron, Davies M. Pfukenyi

**Affiliations:** 1 Department of Clinical Veterinary Studies, Faculty of Veterinary Science, University of Zimbabwe, Mt. Pleasant, Harare, Zimbabwe; 2 Department of Paraclinical Veterinary Studies, Faculty of Veterinary science, University of Zimbabwe, Mt. Pleasant, Harare, Zimbabwe; 3 ASTRE, CIRAD, INRA, Univ. de Montpellier, Montpellier, France; 4 CIRAD, UMR ASTRE, Bangkok, Thailand; 5 Faculty of Veterinary Medicine, Kasetsart University, Bangkok, Thailand; 6 CIRAD, RP-PCP, UMR ASTRE, Maputo, Mozambique; 7 Faculdade de Veterinária, Universidade Eduardo Mondlane, Maputo, Mozambique; Faculty of Science, Ain Shams University (ASU), EGYPT

## Abstract

Anthrax is an important but neglected zoonosis in southern Africa and elsewhere which occurs naturally in herbivorous wildlife and livestock. Fatal outbreaks in animals are spaced by potentially extended periods of non-activity during which the bacterium is maintained in soil. The ecology of the pathogen in the multi-host system and the environment is still not fully understood. This study investigated the patterns of anthrax in Zimbabwe in order to better understand the occurrence of disease in susceptible wildlife and livestock and hence its control. The study used available data in governmental reports between 1995 and 2018 and structured interviewer-administered questionnaires of local communities in three porous wildlife-livestock-human interface sites where livestock/wildlife interactions were documented from previous researches. Two non-interface sites were also included for comparison based on known previous anthrax outbreaks. Respondents from non-interface sites had significantly higher odds (χ^2^ = 23.2, OR = 3.5, 2.1<OR<5.8, p<0.001) of reporting anthrax outbreaks than their counterparts at the interface. Overall 20.0% (74/372) of the respondents reported that some anthrax carcasses were left to dissipate into the environment indicating a risk of environmental contamination. In livestock a total of 214 outbreaks with 2911 losses (mainly cattle) were recorded between 2000 and 2018, while 10 outbreaks with 3171 deaths were noted in wildlife. In humans 99 outbreaks were recorded involving 903 individual cases with 16 fatalities due to enteric infections following the consumption of infected meat between 2010 and 2018. Since its first incidence in wildlife in 2004–2005 in the south-eastern Lowveld of Zimbabwe, anthrax appears to be establishing endemic status along the Zambezi River basin. The disease has expanded spatially affecting 45 (72.6%) of the country’s 62 rural districts in a single decade. Thus, robust multi-disciplinary efforts are encouraged for surveillance and disease containment measures to minimize its impact on livestock, wildlife and humans.

## Author summary

Anthrax is an expanding zoonotic and tropical disease which negatively impacts livestock, wildlife and human health ultimately impacting livelihoods and biodiversity conservation. In this work we have shown it to be a serious disease in Zimbabwe where its surveillance and control are sub-optimum even though it is causing serious losses in animals and human health. Observed practices such as the non-burial of infected carcasses subsequently contaminating the environment following anthrax outbreaks in animals, have most likely contributed to its expansion in geographic range and the increase in frequency of outbreaks. Since the late 1970s, the disease status has changed from that causing a low-level mortality in livestock, probably due to underreporting, to a common disease in livestock, wildlife and humans. It is hoped that by quantifying the impact of the disease across livestock, wildlife and human health and livelihoods, and establishing factors responsible for its continued expansion, adequate resources for surveillance and containment of anthrax will be allocated in order to improve rural livelihoods and also enhance wildlife conservation.

## Introduction

Anthrax, caused by *Bacillus anthracis*, an endospore-forming Gram positive bacterium [1,2] is a cosmopolitan disease that exists since antiquity and has become endemic in some regions of the world. Anthrax is endemic in much of sub-Saharan Africa where it occurs naturally in wildlife and livestock, with herbivores being particularly susceptible [3–6]. Zimbabwe, alongside other southern African countries, falls under anthrax endemic regions [7–11]. In the region, sporadic outbreaks of the disease often cause significant deaths in local populations of both wildlife and livestock in affected foci [8–11]. Despite this, anthrax-related die-offs of wildlife and livestock are poorly documented and thus the disease incidence and impacts are under-reported [12,13]. For various reasons, there is a global under reporting of anthrax in all animals including livestock. The under reporting of anthrax occurrence is higher in wildlife because, unlike livestock, wildlife populations are not closely monitored for disease [7,12,13]. However, some outbreaks may also be missed in livestock because of small numbers of infected and/or isolated infected wild animals often roaming freely and not detected. These also contribute to under-reporting and hence sub-optimal surveillance of the disease in all animals [7,14].

Whenever anthrax epidemics occur in animals, they are often associated with increased incidence of human anthrax, especially in resource-limited communities whose livelihood is largely dependent on livestock production [14,15] or animal resources such as game meat in wildlife-livestock interface areas [16–19]. However, it is not known if livestock/ wildlife interactions at wildlife-livestock-human interfaces (further referred to here as ‘interface’) has any influence on the transmission dynamics of the disease in either species [12,20,21]. In Zimbabwe, no comprehensive epidemiological studies have been undertaken to understand the incidence, propagation and impact of anthrax in animals at the wildlife-livestock-human interface. Hence, most of the available information is either disjointed, scattered in various government agencies or agents or is anecdotal. Clegg *et al*. (2007) [14] reported on a massive outbreak of anthrax in Malilangwe Wildlife Reserve in Chiredzi District of Zimbabwe in 2004 where more than 1500 wild herbivores, and mostly greater kudu (*Tragelaphus strepsiceros*) died. But the report is specifically centred on Malilangwe Wildlife Reserve, yet there is anecdotal evidence that this was only part of a larger outbreak involving other nearby wildlife and livestock areas as ascertained on visits to the area by the principal researcher (2015–2016).

The sub-optimal surveillance, under-reporting and the consequent under-estimation of the disease impact collectively undermine the basis for informed disease containment measures including policy formulation for safeguarding animal and human health, communication and awareness of local extension services and human communities at risk. The epidemiological gaps emanating from inadequate surveillance and documentation of anthrax in all animals and its impact at the interface inspired this work. The specific objectives of this study were therefore: to determine and compare the patterns of anthrax outbreaks in wildlife, livestock and humans at selected interface and non-interface sites in Zimbabwe based on available data, and to determine the knowledge, attitude and practices (KAP) of communities on anthrax to ascertain disease containment measures following an outbreak.

## Materials and methods

### Study areas

**Fig 1:**
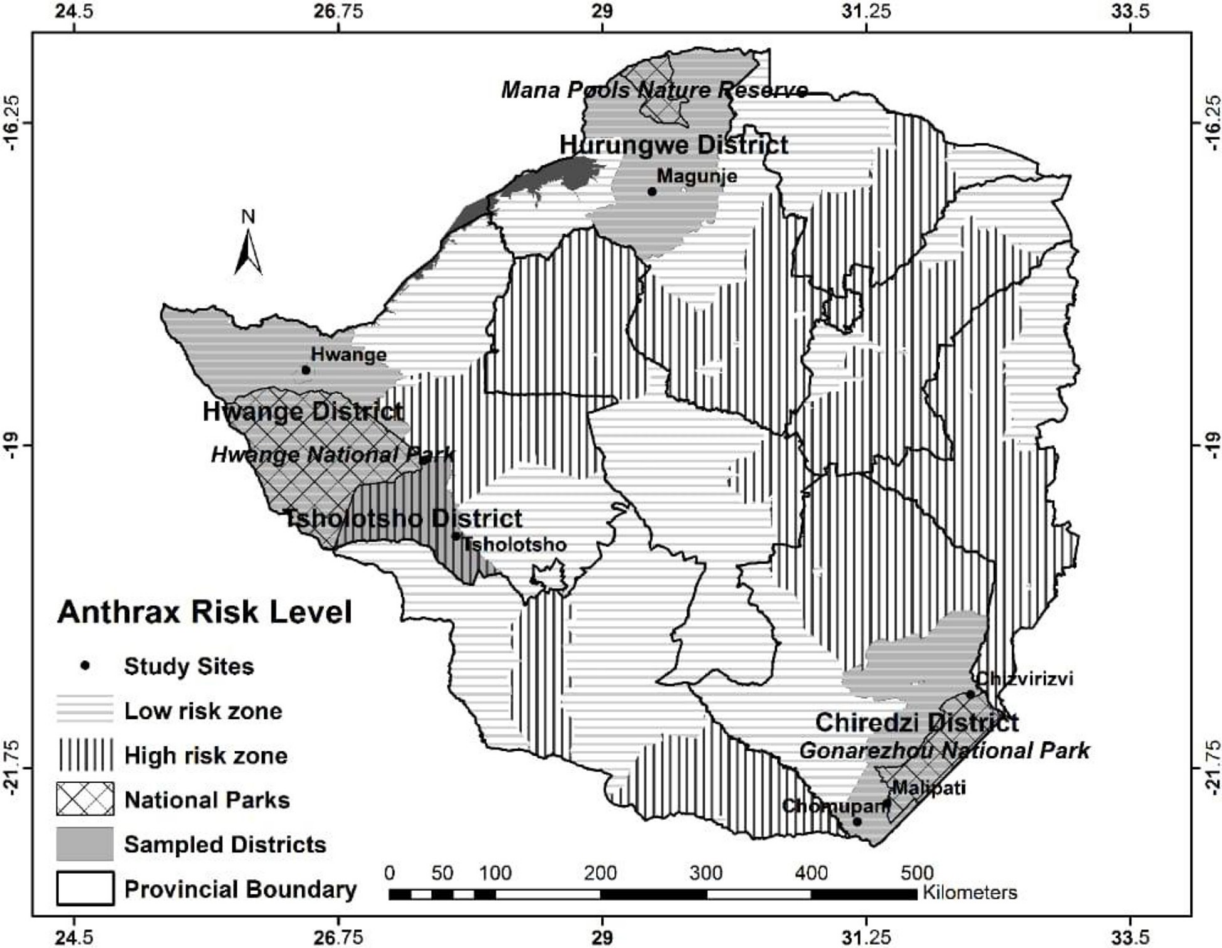
Map of Zimbabwe showing study sites and anthrax risk zones.

The study sites were selected to represent well defined porous wildlife-livestock-human interface and non-interface areas of Zimbabwe as previously identified [22] (Fig 1). The wildlife-livestock-human interface (interface) was defined as an area with a potential overlap between sympatric wildlife, livestock and human populations, either through a direct physical sharing of the same space at the same time or an indirect sharing through sequential contacts with soil, forage and water [21]. Porous interface sites were those adjacent to wildlife areas such as National Parks (NPs) or other protected areas, where there were known interactions between wildlife and livestock. At these sites, livestock were anticipated to interact with wildlife including through predation by wild carnivores. Interaction between sympatric wildlife and livestock at the interface has inherent potential for the sharing of pathogens across species. Non-interface sites were also chosen as sites distant (more than 15 km was considered distant based on maximum daily distance of common wild ungulates) from protected areas where there were no known interactions between livestock and wild animals, directly or indirectly. Hwange and Gonarezhou NPs are respectively the largest and second largest NPs in Zimbabwe, both supporting large wildlife populations and porous interface sites were selected based on their closeness to these two NPS. Four porous interface sites were selected: two close to Gonarezhou NP, Malipati and Chizvirizvi in Chiredzi district and another two adjacent to Hwange NP, Ngamo in Tsholotsho district and Hwange in Hwange district (Fig 1, Table 1). Three non-interface sites, at least 15km from a NP or a protected area were also chosen namely, Chomupani in Chiredzi, Magunje in Hurungwe and Tsholotsho South in Tsholotsho districts, as described in a previous study [22]. Some of the selected study sites had a history of anthrax outbreaks in livestock and in some cases wildlife as well, according to information made available by the Epidemiology and Disease Control Unit, Department of Veterinary Services (DVS), Ministry of Lands, Agriculture, Water, Climate & Rural Resettlement, Zimbabwe.

**Table 1:** Sources of livelihoods according to interface type: (Each interviewee indicated benefiting from one or more source of livelihoods).

Livelihood category	Interface n = 226		Non interface n = 146		Overall n = 372	
	No.	% (95% CI)	No.	% (95% CI)	No.	% (95% CI)
Crop production	214	94.7 (90.7–97.1)	136	93.2 (87.4–96.5)	350	94.1 (91.1–96.2)
Livestock production	206	91.2 (86.5–94.4)	134	91.8 (85.8–95.5)	340	91.4 (88.0–94.0)
Employment other than wildlife	72	31.9 (25.9–38.4)	48	32.9 (25.5–41.2)	120	32.3 (27.6–37.3)
Wildlife based employment:	26	11.5 (7.8–16.6)	0	0.0 (0.1–3.2)	26	7.0 (4.7–10.2)

### Data collection

Anthrax surveillance system for both animal and human sectors in Zimbabwe is based on notification of the disese. The disease is respectively notifiable in animals and humans under the Animal Health and Public Health Acts of Zimbabwe which are in line with respective provisions of Terrestrial Animal Health Code under World Organization for Animal Health (OIE) and International Health Regulation (2005) administered by the World Health Organization (WHO). Hence, it is mandatory that when anthrax is suspected in animals or humans the appropriate animal or human health authorities are notified. The disease is then confirmed and appropriate control measures are taken. Epidemiological information about anthrax outbreaks in animals and humans is also transmitted to OIE and WHO through the animal and human health authorities, respectively.

Anthrax outbreaks were identified and defined by their spatio-temporal distance; that is separated by their locality and/or time. This was also cross-checked based on the expertise of veterinary and human medical staff who directly followed outbreaks of the disease. Triangulation, the use of two or more data sources was used in this study to collect relevant data. This allowed cross-checking of reports or data provided and the methods used included questionnaires, perusing government records, conducting interviews and focus group discussions and literature search.

A structured interviewer-administered questionnaire was used in order to obtain baseline data on the important sources of livelihoods of livestock owners and the livestock owners’ awareness of anthrax and their anthrax management practices. Sources of livelihoods of the respondents were defined as their means of securing the necessities (e.g. food or income) for general sustenance of life. The questionnaire was pre-tested on 15 randomly selected farmers from a different area than the study sites in order to assess the clarity of the questions and the time needed to administer the questionnaire. The completion easiness of the questionnaire, redundant sections and lack of clarity of some questions were noted and later revised to improve accuracy of the data collected. The selection of the final respondents in the study sites was based on voluntary oral consent and the method was already described in detail in Mukarati *et al*. (2018) [22]. Briefly, on average each site is made up of about 30 villages and each village has 30 households, giving an average of 900 households per site and 6300 households across the seven sites. The sample size of households to be sampled per site was calculated using the formula: *n* = [*z*^2^ x *p* (1-*q*)]/*e*^2^, where *z* = 1.96 for 95% confidence interval, *p* = estimated prior knowledge of anthrax (prevalence) in the communities based on other studies, and *e* is the desired precision. We estimated the prior knowledge of anthrax in the communities to be at 71.5% based on previous studies conducted in similar rural communities [23], and a 10% error margin at 95% confidence level. This estimated a minimum of 73 households per site to be therefore targeted for sampling. But based on resources available, approximately 50 (5.6%) households per site were selected by both simple and systematic random sampling procedures. In each site, the first sampling unit was a livestock-owning household selected. Thereafter and by systematic random sampling, every third or next livestock-owning household was included in the survey and the process repeated till the targeted number of interviewees per site was reached. From each of the selected households, the heads or their representatives were interviewed by the principal investigator individually for approximately 20–30 min and responses recorded. The local animal health extension officers in their respective areas assisted the principal investigator in conducting the interviews including interpretation in vernacular languages (Shona, Shangani, Ndebele & Nambiya) depending on the interviewees’ preferences.

In the questionnaire, the information collected included the respondents’ demographic characteristics and their sources of livelihoods (crop agriculture, livestock rearing or both). The interviewees were asked on anthrax awareness, anthrax outbreaks in livestock, wildlife and humans in their respective areas, livestock and wildlife species affected, the respondents’ and responsible authorities anthrax management practices in both livestock and wildlife, detailed history of anthrax outbreaks in the last ten years before 2015, individual households’ losses of livestock during the episodes of anthrax outbreaks and human anthrax cases accompanying outbreaks in livestock and wildlife.

The secondary information on human anthrax outbreaks and cases was obtained from the Department of Epidemiology and Disease Control, Ministry of Health & Childcare, Zimbabwe while that of livestock and wildlife was collected from the DVS. A human anthrax case was defined as any person exposed to an infected animal or materials and presenting with typical cutaneous, gastrointestinal, pulmonary or other signs of anthrax and confirmed through either isolation of *B*. *anthracis* from an affected tissue or other supportive laboratory tests [24]. In livestock and wildlife, anthrax confirmation was based on clinical signs and demonstration of blue-staining bacillus rods surrounded by a pink staining capsule on blood smears stained with polychrome methylene blue and examined under light microscopy (the MacFadyean reaction) [25]. The outbreaks and cases described in this study were all laboratory confirmed.

The data extracted from reported human anthrax cases for a period of 8 years (2011–2018) included district name, number of cases, type of cases (cutaneous, enteric or pulmonary), clinical outcome of the cases (recovery or death). The extracted human data was aggregated and anonymized with no identification details of the affected patients. Data on livestock and wildlife anthrax outbreaks and cases for the study sites and at a national level was obtained for a period of 24 years (1995–2018) from the DVS records.

Additionally, local information regarding anthrax in wildlife wasgathered during on-site focus group discussions (FGDs) with key wildlife informants of the Zimbabwe Parks and Wildlife Management Authority, Communal Areas Management Program for Indigenous Resources (CAMPFIRE Program) as well as wildlife safari operators and private game parks, such as Malilangwe Wildlife Reserve in Chiredzi district of Zimbabwe. The key informants were selected on the basis of their knowledgeof wildlife and field experience in the study interface areas. Each category of the key informants, comprised of 8–10 members was interviewed separately and the data was collected through audio tapes and notes by a facilitator and the principal investigator. The FGDs were conducted as a means of verifying some of the secondary data obtained, especially cross-checking dates/sites of anthrax outbreaks.

### Data analysis

The data was captured in Microsoft Excel 97–2003 where data edits were performed and then transferred to the Software Package for Social Sciences (SPSS) version 16 and STATA SE/11 to generate descriptive statistics (frequencies/proportions). The questionnaire data were analysed with respect to the respondents’ areas of origin (sites), their sources of livelihood and the proportion reporting anthrax outbreaks (0 = no; 1 = yes) according to the type of interface (0 = interface; 1 = non-interface). Explanatory variables used were the area of origin (”site”) and the type of interface (“interface” or “non-interface” areas). The Yates corrected Chi-squared test and odds ratio (OR) were used to assess the relationship between explanatory variables presented above and the questionnaire’ responses (e.g. number of respondents reporting or not reporting anthrax outbreaks). Associations were evaluated at both the site and interface levels and were considered significant at p< 0.05. The qualitative data generated during FGDs was tabulated and the responses were categorized and then converted to quantitative data before being analyzed. This data included location, year of outbreaks, main species affected and approximate mortality numbers, any spillover to or from livestock and humans, management of the outbeaks by animal health authorities and the communities, whether preventive vaccination programs were in place and followed and any other information volunteered by the FGDs participants. Descriptive statistics with respect to main points raised by the respondents from the different interface sites were then generated from the FGDs quantitative data.

Anthrax outbreaks in livestock and wildlife from 1995 to 2018 were further tabulated with respect to the total number of outbreaks and cases in the respective areas. Data on mortalities attributed to the disease were tabulated yearly (2000–2018) according to species (livestock, wildlife and humans) to provide the temporal patterns of anthrax in different species.

Geo-referenced data made available by DVS and Ministry of Health and Child Care was used to create spatial distribution maps for outbreaks of anthrax in animals (livestock and wildlife) and humans. The maps were created using DIVA -GIS software (www.diva-gis.org/gdata). Anthrax outbreak points were overlaid on shapefiles of administrative provinces, districts and wildlife conservation areas of Zimbabwe downloaded from www.diva-gis.org/gdata. These maps were grouped into two periods: 2000 to 2009 and 2010 to 2018 to show trends in spatial distribution of anthrax outbreaks in livestock and wildlife over time. The distribution map for human cases was done for 2011–2018 according to available data.

### Ethical considerations

Ethical approval for the use of data records and for all protocols used in this study was obtained rrspectively from the Higher Degrees and Ethics Committees of the Faculty of Veterinary, University of Zimbabwe, DVS, Ministry of Health and Child Care and the Zimbabwe Parks and Wildlife Management Authority. The purpose of this study was well explained to all the livestock owners and key informants who participated in this study on orally expressed self consent.

## Results

The study sites representing well defined wildlife-livestock-human interface and non-interface areas of Zimbabwe are depicted in Fig 1.

### Demographic characteristics of respondents

A total of 372 respondents were interviewed with 60.8% (226/372) of them originating from the interface sites. Females comprised 64.5% (240/372) of the respondents. Of the total respondents, 87.6% (326/372) were adults aged 30 years or more while the other 12.4% of respondents were younger than 30 years but older than 18 years.

### Livelihoods and sources of income

Overall, the respondents relied on crops (94.1%) and livestock rearing (91.4%) for their livelihoods across the interface and non-interface areas. Some also benefited from formal employment (32.3%) (Table 1). Employment in wildlife related activities (11.5%) was restricted to respondents from the interface sites.

About 46.9% of the respondents from interface sites benefited from other forms of exploitation of wildlife areas such as accessing grazing areas and water for livestock (16%, 36/226) and also harvesting natural products (9.7%, 22/226) which included thatching grass, firewood, wild fruits, mopane worms (*Imbrasia belina*; *Amacimbi* in Ndebele) and others. Respondents from interface sites reported prevalence of human-wildlife conflicts such as crop destruction and livestock predation (80.5%; 182/226), the presence of infectious diseases hampering profitable livestock production (58.4%; 132/226) and loss of livestock markets due to infectious diseases (46.0%; 104/226), such as foot and mouth disease, buffalo-derived theileriosis (Corridor disease) among other pathogens which are spread from wildlife to livestock [26].

### Respondents reporting presence of anthrax in their areas

A summary statistics of respondents reporting and not reporting anthrax outbreaks according to site and interface type is shown in Tables 2 and 3. Overall, 68.8% of the respondents reported some experience with anthrax outbreaks in animals. The overall percentage of respondents from the non-interface sites reporting anthrax outbreaks was significantly higher (*p* < 0.05) than that from the interface sites. The non-interface sites were associated with respondents having a significantly higher odds (χ^2^ = 23.2, OR = 3.5, 2.1<OR<5.8, p<0.001) to report anthrax outbreaks than their counterparts from the interface sites. The proportion of respondents reporting anthrax outbreaks varied significantly (p<0.05) among sites. Malipati and Chomupani sites in the interface and non-interface areas respectively, recorded significantly (p<0.05) lowest proportions of respondents reporting anthrax outbreaks compared to other sites in their respective areas. In the interface area, Chizvirizvi site had the highest (88.5%) proportion and was associated with respondents having a significantly higher odds (χ^2^ = 43.0, OR = 21.3, 7.8<OR<58.3, p<0.001) to report anthrax outbreaks while Magunje site had the highest frequency of anthrax report (100%, χ^2^ = 70.4, p<0.001) among all the non-interface sites.

**Table 2:** The number and per cent of respondents reporting and not reporting anthrax outbreaks in animals according to interface type and site.

		Number reporting anthrax				
		YES		NO		Total
Category		Number (%)	95% CI	Number (%)	95% CI	
Interface		134 (59.3^b^)	52.6–65.7	92 (40.7)	34.3–47.4	226
Non-interface		122 (83.6^c^)	76.3–89.0	24 (16.4)	11.0–23.7	146
**Total**		**256 (68.8)**	**63.8–73.4**	**116 (31.2)**	**26.6–36.2**	**372**
	Site					
Interface	Malipati	18 (26.5^a^)	16.8–38.8	50 (73.5)	61.2–83.2	68
	Hwange	32 (61.5^b^)	47.0–74.4	20 (38.5)	25.6–53.0	52
	Ngamo	38 (70.4^b^)	56.2–81.6	16 (29.6)	18.4–43.8	54
	Chizvirizvi	46 (88.5^cb^)	75.9–95.2	6 (11.5)	4.8–24.1	52
Non-interface	Chomupani	4 (20.0^a^)	6.6–44.3	16 (80.0)	55.7–93.4	20
	Tsholotsho South	38 (82.6^c^)	68.1–91.7	8 (17.4)	8.3–32.0	46
	Magunje	80 (100^d^)	-	0 (0.0)	-	80

Figures with different superscripts for sites and interface type are significantly different at p < 0.05

**Table 3:** Summary statistics of respondents reporting and not reporting anthrax outbreaks in animals according to interface type and site.

Category		% Reporting anthrax	% Not reporting anthrax	*OR	95% CI	Yates Corrected X^2^	p-value
Interface		59.3	40.7	-	-	-	-
Non-interface		83.6	16.4	3.5	2.1–5.8	23.2	0.0000
	Site						
Interface	Malipati	26.5	73.5	-	-	-	-
	Hwange	61.5	38.5	4.4	2.1–9.7	13.5	0.0002
	Ngamo	70.4	29.6	6.6	3.0–14.6	21.6	0.0000
	Chizvirizvi	88.5	11.5	21.3	7.8–58.3	43.0	0.0000
Non-interface	Chomupani	20.0	80.0	-	-	-	-
	Tsholotsho South	82.6	17.4	19.0	5.0–72.2	21.0	0.0000
	Magunje	100.0	0.0	-	-	70.4	0.0000

*A site with the lowest percentage value was taken as the reference for calculating the odds ratio (OR) and similarly the interface type with the lower percentage value was taken as the reference for calculating OR.

### Respondents’ knowledge and practices on anthrax

Table 4 shows anthrax knowledge and management practices by respondents based on past disease outbreaks. Knowledge of anthrax varied, with a significantly (p<0.001) higher percentage (67.1%) of respondents from the non-interface being aware of human anthrax cases compared to those from the interface sites (10.6%). Some respondents (7.8%) indicated that anthrax was detected in livestock as a trace back from human cases while others (17.7%) also indicated that carcasses of livestock dying from anthrax were salvaged for human consumption.

**Table 4:** Summary of respondents’ knowledge and practices on prevention and management of anthrax in livetsock according to interface and non-interface areas.

Variable	Responses	Interface (n = 226)		Non-interface (n = 146)		Overall (n = 372)	
		No.	% (95% CI)	No	% (95% CI)	No	% (95% CI)
Can 00a0confidently recognize signs of anthrax in animals	Yes	62	27.4^a^ (21.8–33.8)	48	32.9^a^ (25.5–41.2)	110	29.6 (25.0–34.5)
Aware of human anthrax cases in general	Yes	24	10.6^a^ (7.1–15.6)	98	67.1^b^ (58.8–74.5)	122	32.8 (28.1–37.9)
Aware of human anthrax cutaneous cases	Yes	22	9.7^a^ (6.3–14.6)	98	67.1^b^ (58.8–74.5)	120	32.3 (27.6–37.3)
Aware of human anthrax enteric cases	Yes	2	0.9 (0.2–3.5)	0	0.0 (0.1–3.2)	2	0.5 (0.1–2.1)
Diagnosis confirmed in animals	Yes	80	35.4^a^ (29.3–42.1)	94	64.4^b^ (56.0–72.0)	174	46.8 (41.6–52.0)
Disease detected in animals as trace-back from human cases	Yes	13	5.8^a^ (3.2–9.9)	26	17.8^b^ (12.2–25.2)	29	7.8 (5.4–11.1)
Carcass disposed by burning / burying	Yes	96	42.5^a^ (36.0–49.2)	56	38.4^a^ (30.5–46.8)	152	40.9 (35.9, 46.1)
Carcass salvaged for human consumption	Yes	40	17.7^a^ (13.1–23.4)	26	17.8^a^ (12.2–25.2)	66	17.7 (14.1, 22.1)
Carcass left to dissipate into the environment	Yes	62	27.4^a^ (21.8–33.8)	12	8.2^b^ (4.5–14.2)	74	20.0 (16.0, 24.4)
Uncertain how carcasses were disposed off	Yes	28	12.4^a^ (8.5–17.6)	30	20.6^a^ (14.5–28.2)	58	15.6 (12.1–19.8)
Routine/regular vaccination of animals	Yes	182	80.5^a^ (74.6–85.4)	62	42.5^b^ (34.4–50.9)	244	65.6 (60.5–70.4)
Vaccination in face of outbreaks only	Yes	92	40.7^a^ (34.3–47.4)	88	60.3^b^ (51.8–68.2)	180	48.4 (43.2–53.6)
Will not report to veterinary authorities when anthrax suspected	Yes	40	17.7^a^ (13.1–23.4)	22	15.1^a^ (9.9–22.2)	62	16.7 (13.1, 20.9)

Values with similar superscripts in the same rows are not significantly different (p>0.5).

Respondents’ knowledge of best practices for anthrax prevention is shown in Table 4. Over 60% of the interviewed respondents were aware of regular vaccination as an anthrax preventive measure while others (40.9%) were also aware of proper burning/burial being a preventive measure against future outbreaks. Less than a quarter of the respondents (15.6%) were unaware of proper disposal methods while 20% indicated that anthrax carcasses were left unburied to dissipate into the environment. For various reasons, which include disillusionment with non-attendance by extension workers to reported animal health cases, the desire to salvage some value from sick animals through emergency slaughtering and selling the meat, among others, less than a fifth (16.7%) of the respondents indicated that they do not report suspected anthrax outbreaks to the veterinary authorities. It was further indicated that the sequence of response to outbreaks of anthrax by communities was: initially salvaging carcasses of dead or hastily slaughtered sick livestock for human consumption, followed by burial or burning of some carcasses and, finally efforts at disposal and environmental decontamination abandoned and carcasses left to dissipate into the environment as carcass numbers increased.

### Management of wildlife anthrax outbreaks

Information from governmental records and FGDs indicated that for all anthrax outbreaks noted in wildlife in this study (*n* = 10 documented and cross referenced), only Malilangwe Wildlife Reserve had a history of carcass disposals and environmental decontamination efforts such as burning or burying carcasses during an outbreak in 2004–2005. For all other outbreaks of anthrax in wildlife, only disease confirmation was done as already described, but with no containment measures undertaken.

### Recorded anthrax outbreaks in Zimbabwe

A total of 45 anthrax outbreaks were recorded in the study sites during the period 1995–2018 with approximately 82.2% (37/45) of them being from livestock (Tables 5 and 6).

**Table 5:** Recorded anthrax outbreaks* and cases in livestock in study sites (1995–2018).

Category				
Interface	Site	Year	Outbreaks	Cases
	Hwange	1995	5	11
	Ngamo	-	1	6
	Chizvirizvi	2004	1	10
		2006	2	20
		2008	3	45
		2009	2	30
		2010	1	9
	**Sub-total**		**15**	**131**
**Non-interface**	Tsholotsho South	2000	1	15
		2010	2	25
		2011	2	30
		2012	2	20
		2015	3	40
		2017	3	36
	Magunje	2001	1	10
		2005	1	15
		2006	1	9
		2009	1	12
		2010	1	17
		2014	1	29
		2015	1	18
		2016	1	21
		2017	1	38
	**Sub-total**		**22**	**335**
	**Grand total (cattle)**		**37**	**466**

* An anthrax outbreak was the occurrence of one or more animal deaths at a particular place and time which was laboratory confirmed to have been infected with *B*. *anthracis*.

**Table 6:** Recorded anthrax outbreaks and cases in wildlife in study sites (1995–2018).

Category				
Interface	Site	Year	Outbreaks	Cases
	Malilangwe Wildlife Reserve	2004	2	2822
	Mana Pools National Park*	2011	1	68
		2015	1	75
		2018	1	69
	Zambezi National Park	2015	2	10
		2017	1	6
	**Total**		**8**	**3050**

*Also called Mana Pools Nature Reserve.

A total of 3516 anthrax cases were recorded in animals at the study sites, with wildlife (86.7%, 3050/3516) contributing a higher percentage of the cases than livestock (Tables 5 & 6). However, the frequency of anthrax outbreaks was higher in livestock than in wildlife. In livestock, cattle anthrax outbreaks contributed 94.6% of the total (35/37); one outbreak was in goats while another was in the indigenous free-ranging domestic pigs (Mukota breed). Livestock anthrax outbreaks at non-interface sites contributed 59.5% (22/37) of the outbreaks.

During the period under review (1995–2018), anthrax outbreaks were first recorded in 1995 for livestock and in 2004 for wildlife. Incidentally, the 2004 anthrax incident was the very first recorded outbreak of the disease in wildlife in Zimbabwe. The years of anthrax outbreaks and species involved are summarized in Tables 5 and 6. Two outbreaks of anthrax in humans were reported in the non-interface area; one in Tsholotsho South and another one in Magunje, respectively attributed to cases in goats and pigs.

At the national level, between 2000 and 2018, a total of 214 outbreaks of anthrax were recorded in livestock, with losses of 2911 animals (mostly cattle), while 10 outbreaks with 3171 deaths were recorded in wildlife (Table 7).

**Table 7:** National livestock and wildlife deaths and human cases of anthrax based on reported disease outbreaks in Zimbabwe 2000–2018.

YEAR	Wildlife deaths			Livestock deaths		Humans clinical cases & deaths		
	Site	No. of outbreaks	No. of cases	No. of outbreaks	No. of cases	No. of outbreaks	No. of clinical cases	No. of deaths
2000	-	-	-	6	123	-	-	-
2001	-	-	-	19	217	-	-	-
2002	-	-	-	7	70	-	-	-
2003			-	12	169	-	-	-
2004	Malilangwe Wildlife Reserve	2	2822^K^	23	450	-	-	-
2005	-	- -	-	24	628	-	-	-
2006	-	-	-	20	312	-	-	-
2007	-	-	-	0	0	-	-	-
2008	-	-	-	2	4	-	-	-
2009	-	-	-	10	82	-	-	-
2010			-	11	55	-	-	-
2011	Mana Pools Natinal Park	1	160^H^	8	34	-	-	-
2012	-	-	-	13	238	7	34	7
2013	-	-	-	12	33	20	225	6
2014	-	-	-	12	113	20	77	0
2015a	Zambezi National Park	2	16^B, E^	10	146	21	224	1
2015b	Chirundu Safaris, Zambezi Valley	1	B					
2016			-	16	131	22	230	1
2017	Zambezi National Park & Binga	1	16^B, H^	12	75	9	57	1
2018a	Mana Pools National Park	2	155^I^	9	31	-	56	0
2018b	Leopard’s Rock, Mutare	1	2^Z^					
**Total**		**10**	**3171**	**214**	**2911**	**99**	**903**	**16**

Letter superscripts represent primary species affected in respective anthrax outbreaks: K–greater kudu (*Tragelaphus strepsiceros)*, E- elephant (*Loxodonta africana*), H–Hippos (*Hippopotamus amphibious*), B- African buffalo (*Syncerus caffer*), I–impala (*Aepyceros melampus)*, Z- zebra *(Equus burchelli)*.

**Fig 2:**
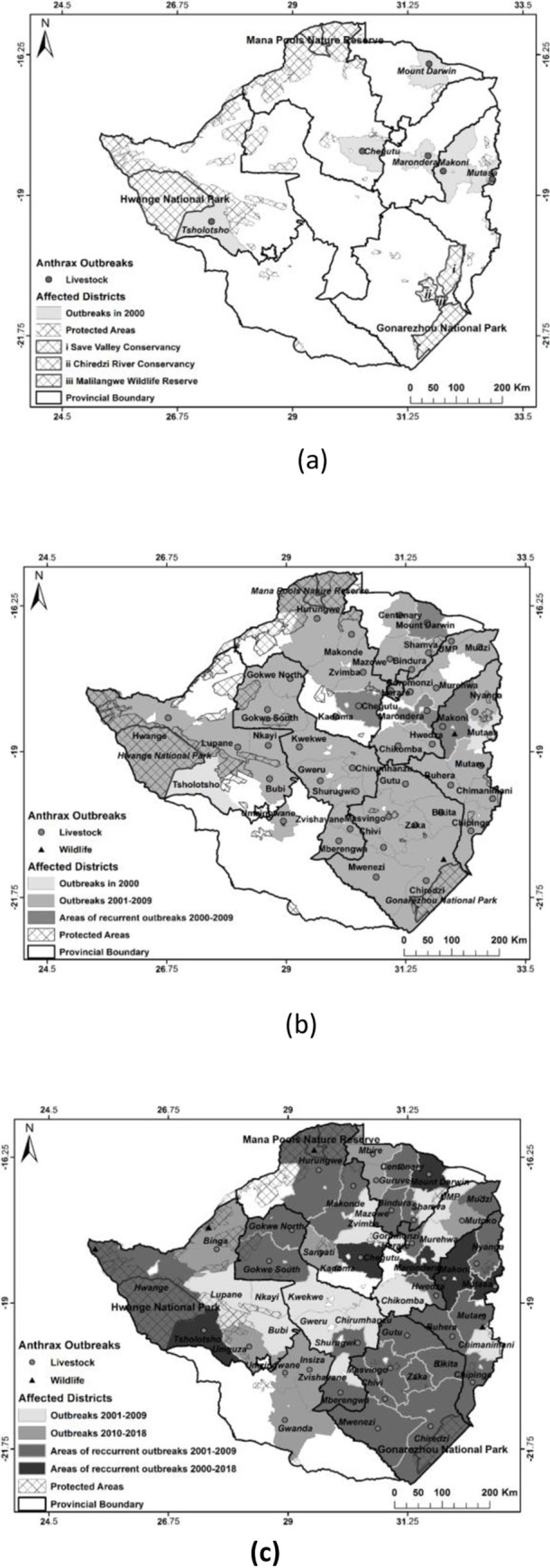
Spatial distribution of anthrax outbreaks in livestock and wildlife 2000 (a), 2001–2009 (b) & 2010–2018 (c).

The spatial distribution of anthrax outbreaks in livestock and wildlife are given in Fig 2.

### Wildlife anthrax outbreaks: Temporal trends and primary species affected

From the 2004–2005 first massive outbreak in wildlife in Malilangwe Wildlife Reserve and Save Valley Conservancy in the south-eastern Lowveld of Zimbabwe, anthrax has been occurring almost every year especially along the Zambezi River basin from Zambezi National Park (Kazungula, west of Victoria Falls) to Mana Pools National Park in the mid-Zambezi area (Table 7). The major species affected along the Zambezi River basin were primarily hippos and buffaloes while other species were affected to a lesser extent including elephants and impalas.

Anthrax has been diagnosed in a total of 31 identified wildlife species belonging to 13 families in Zimbabwe (Table 8). The Bovidae family was mostly affected. Further, an affected jackal and mongoose were not identified to species level.

**Table 8:** List of wild animal species in which anthrax infection has been reported in Zimbabwe.

Family	Common name	Scientific name	References
Bovidae	African Buffalo	*Syncerus caffer*	[14,27]
	Blue Wildebeest	*Connochaetes taurinus*	[14,27,28]
	Bushbuck	*Tragelaphus scriptus*	[14,28]
	Common Duiker	*Sylvicapra grimmia*	[14]
	Common Eland	*Taurotragus oryx*	[14,28]
	Common Reedbuck	*Redunca arundinum*	[14,28]
	Greater Kudu	*Tragelaphus strepsiceros*	[14,28]
	Impala	*Aepyceros melampus*	[14,27,28]
	Klipspringer	*Oreotragus oreotragus*	[14,28]
	Nyala	*Tragelaphus angasii*	[14,28]
	Roan Antelope	*Hippotragus equinus*	[14,28]
	Sable Antelope	*Hippotragus niger*	[14,28]
	Sharpe’s Grysbok Waterbuck	*Raphicerus sharpie*	[14]
		*Kobus ellipsiprymnus*	[14,28]
Canidae	*Jackals	(*Species* not identified)-	[27]
	Wild dog	*Lycaon pictus*	[14,28]
Cercopithecidae	Chacma Baboon	*Papio ursinus*	[14,28]
Elephantidae	African Elephant	*Loxodonta Africana*	[27]
Equidae	Common Zebra	*Equus burchelli*	[14,28]
Felidae	Caracal	*Caracal caracal*	[14]
	Cheetah	*Acinonyx jubatus*	[14]
	Leopard	*Panthera pardus*	[14,27,28]
	Lion	*Panthera leo*	[14]
Giraffidae	Giraffe	*Giraffa camelopardalis*	[14,27,28]
Herpestidae	*Mongoose	(*Species* not identified)-	
	Banded Mongoose	*Mungos mungo*	[14]
Hippopotamidae	Hippopotamus	*Hippopotamus amphibious*	[14,28]
Hyaenidae	Spotted hyena	*Crocuta crocuta*	[14]
Orycteropodidae	Aardvark	*Orycteropus afer*	[27]
Suidae	Bush pig	*Potamochoerus larvatus*	[14,28]
	Common Warthog	*Phacochoerus africanus*	[14,28]
Viverridae	African Civet	*Civettictis civetta*	[27]
	Common Genet	*Genetta genetta*	[27]

*Jackals and mongooses were not identified to species level.

### Anthrax cases in humans

**Fig 3:**
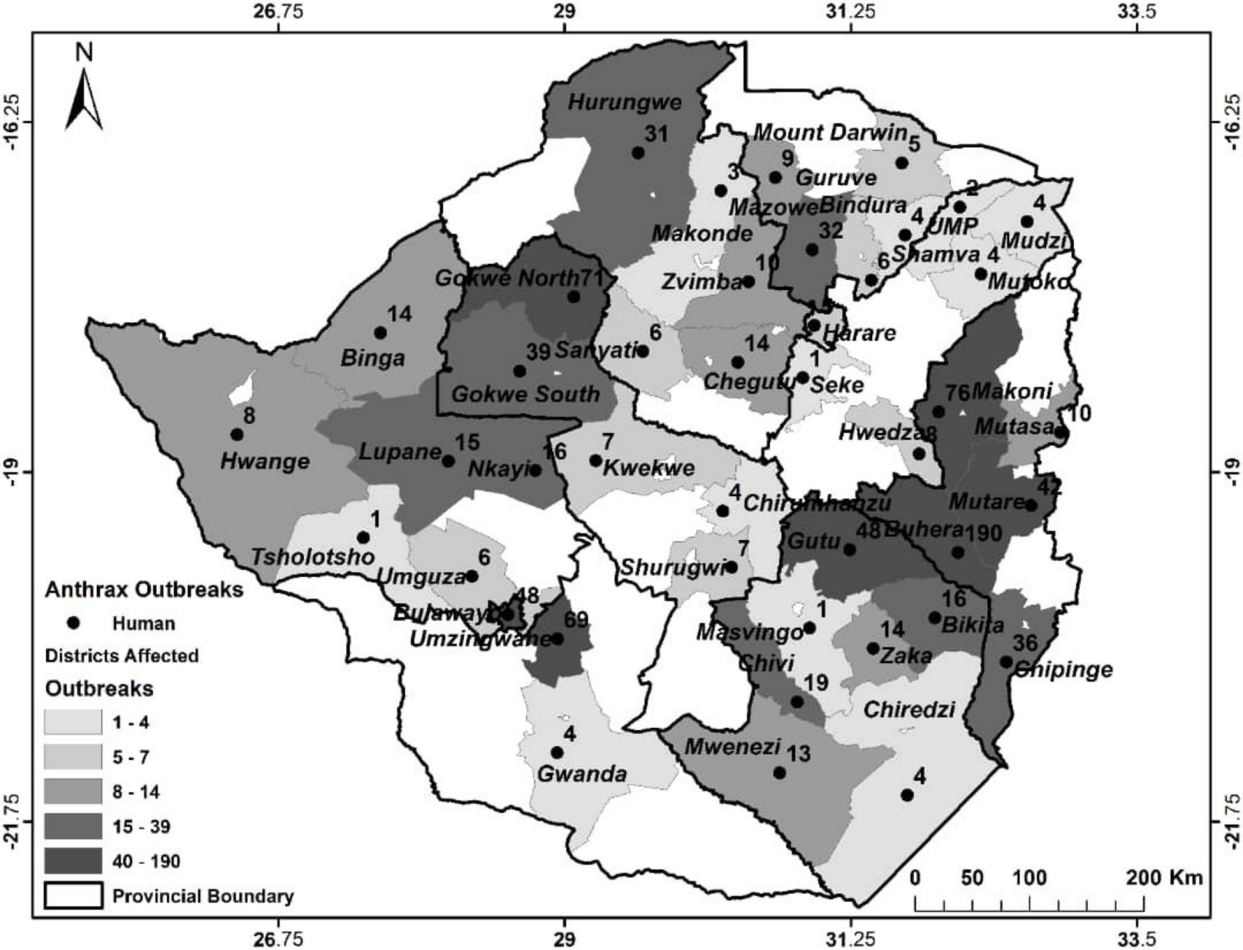
Spatial distribution of anthrax cases in humans between 2010 and 2018.

Between 2010 and 2018 there were 99 outbreaks of anthrax in humans with 903 cases of which 16 (1.8%) were fatal (Table 7) and almost all but one due to enteric form of the disease. Cutaneous anthrax accounted for 98.3% (888/903) of human cases. There were no pulmonary or other forms of anthrax diagnosed in humans under this study. The human cases of anthrax were also widely distributed across 45 districts (Fig 3) with no apparent geographical concentration. Further, human cases were reported across the officially recognized high and low risk zones for anthrax outbreaks. According to the Department of Epidemiology & Disease Control, Ministry of Health & Child Care (Zimbabwe) all human cases were secondary to the disease in livestock through handling or consumption of meat from infected carcasses with the latter route of infection resulting in enteric disease. In some cases the disease in animals was only noted restrospectively as trace back from the human cases.

## Discussion

Anthrax is present across most of Zimbabwe’s territory and ecosystems, with an increasing trend in spatial coverage in recent years. There has been an increase in both range and frequency of the disease in livestock, wildlife and humans. The increasing trend was clearer in wildlife where since the first recorded outbreak in 2004 [14], anthrax has being reported with increasing frequency in free-ranging wildlife, especially along the Zambezi River basin where it is suspected to be establishing endemicity. Though fewer, outbreaks of the disease in wildlife were accompanied by higher numbers of cases per outbreak than in livestock. The lack of carcasss disposals and environmental decontamination following the disease outbreaks especially in wildlife as noted in this study, are most likely factors contributing to the expansion in the range and frequency of the disease in animals. There is therefore potential for exponential increase in anthrax incidence in wildlife with spillover to in contact livestock and humans. Ultimately, such unchecked wildlife anthrax outbreaks may have serious negative impacts on biodiversity conservation and the related ecotourism industry.

The occurrence and suspected establishment of endemicity of anthrax in wildlife in Zimbabwe along the Zambezi River basin as noted in this study should not be surprising considering that the disease has long been documented as endemic in adjacent parts of neighbouring Zambia [4,29,30]. Thus the increase of anthrax incidence in wildlife on the Zimbabwean side of the Zambezi River basin could be representing a shared disease across an international boundary. However, the factors responsible for the upsurge of the disease in wildlife on the Zimbabwean side are not clear and need further investigation.

On the other hand and with regard to reasons for spontaneous outbreaks of anthrax in animals, Van Ness (1971) [11] postulated the occurrence of ‘anthrax incubator areas’ in which *B*. *anthracis* spores are suspected to be concentrated in regressing water bodies to sufficient levels so as to initiate an outbreak during the dry seasons. This ‘anthrax incubator areas’ postulation may possibly explain in part the observed anthrax outbreaks in hippopotamus along the Zambezi River. It was established that outbreaks occurred when the water in the Zambezi River had regressed to unusually low levels as noted for anthrax in hippos in Mana Pools National Park in 2011. Nutritional deprivation for all animals in the park was also evident and wildlife rangers reported finding one hippo carcass with a considerable amount of river sand in the stomach. Further, Turner *et al*. (2014) [31] reported on the fatal attraction of herbivores to lush vegetation which is also laden with *B*. *anthracis* spores. Lush vegetation is an obvious attraction for grazers in the presence of nutritional deprivation as reported here in the Zambezi Valley and Mana Pools National Park at the time of anthrax outbreak in the hippos. Hence, based on these two possible mechanisms, timely sampling of suspect small water pools and plants for *B*. *anthracis* could be helpful in the investigation of future outbreaks of anthrax in these riverine areas [31,32].

In agreement with other reports in literature, the wild ungulates in the family Bovidae, including the greater kudu, the buffalo, the bushbuck (*Tragelaphus scriptus*), among others have predominated in mortalities due to anthrax [3,13,33]. In the Save River Conservancy and Malilangwe Wildlife Reserve, the greater kudu accounted for 75.9% and 64.5% of the mortalities, respectively [14]. In total, 28 species of animals have been confirmed to have been affected by anthrax in Zimbabwe. This is expected to rise as elsewhere and Smith *et al*. (2017) [3] gave a global number of 58 species of animals affected by anthrax.

Anthrax is a notifiable disease [6,19] in Zimbabwe and animal health authorities are mandated to coordinate carcass disposal and environmental decontamination following the disease outbreaks as provided for in legislation such as Statutory Instrument 289, Animal Health (General) Regulations, 1994 [34]. However, the animal health authorities are hampered by inadequate resources in enforcing regulations, while communities do not always report suspected cases of anthrax outbreaks in their livestock, but instead knowingly consume infected meat as found in this study. The communities’ actions are influenced by poverty and fear of losses as noted before [16,23,35]. In wildlife, this inadequacy in anthrax control is supported by the observation that for all wildlife anthrax incidents reported here, no carcass disposal or environmental decontamination were done except for the 2004–2005 outbreak in Malilangwe Wildlife Reserve [14]. The shortcomings in disease control efforts noted here are likely to have contributed to the upsurge of anthrax from a disease causing low-level mortality in livestock annually prior to 1978 [36] to the present status where high mortalities are recorded annually in animals and humans (Tables 6 & 7). The expansion in the range of anthrax goes beyond the anthrax high risk zones recognized earlier by the animal and human health authorities and other researchers [24,37]. Clearly, this calls for concerted efforts at mobilization of resources and multi-disciplinary stakeholders’ collaboration under the One Health banner [15,20,38] for effective surveillance and control of anthrax in animals and humans. Given that respectively over 60% and 41% of the respondents were aware of vaccination and burning/burial as anthrax preventive measures, enforcing and more education on these is likely to have a positive impact in preventing anthrax outbreaks in the country.

Although recall bias was one of the main limitations of this study, the significantly higher number of reported anthrax outbreaks by respondents from non-interface than interface sites is corroborated by secondary data of recorded outbreaks by DVS and Zimbabwe Parks and Wildlife Management Authority. For instance, DVS records showed that over 60% of the anthrax outbreaks were from the non-interface sites. It was also apparent that anthrax was not strictly associated with the wildlife-livestock-human interfaces but could be shared between in-contact livestock and wildlife. However, lack of anthrax strain typing data in this study is a limitation with respect to conclusions on the sharing of anthrax between livestock and wildlife. It was also evident that farmers were more acquainted with anthrax in livestock in non- interface areas where it had long been established [36].

Hwange NP shares a diffuse interface with the surrounding Tsholotsho and Hwange communal lands and these communities graze their livestock in adjacent parts of the protected area as corroborated by respondents from interface sites in this study. Predation of livestock in the adjacent communal lands by wild carnivores from the park is well documented [39] further indicating animal species interactions through a porous interface. In this setup, anthrax outbreaks have been reported in livestock butnone in wildlife in adjacent Hwange NP up to 2018. This suggests that either the disease was occurring in Hwange NP wildlife but being missed due to sub-optimal surveillance [40] as ascertained by the principal researcher on area visit (September, 2015), or that it was truly absent due to environmental influences [41,42]. Even though Chikerema *et al*. (2013) [37] placed Hwange district encompassing Hwange NP and the surrounding communal areas in a low risk zone for anthrax outbreaks, the apparent complete absence of anthrax cases in wildlife of this NP requires further investigations and also calls for improved surveillance of the disease.

The occurrence of anthrax as isolated single cases or loss of small numbers of animals at a time in both wildlife and livestock is worth noting. Traditionally, anthrax has generally been considered to be associated with mass mortalities of susceptible species at any one outbreak [5,8,30,43]. However, as noted in this study, mass mortality of animals during outbreaks of the disease was not always the rule. There were isolated cases of anthrax in buffaloes in Zambezi National Park in 2015 where carcasses were opened for post mortem examination because the disease was not suspected from the pattern of mortality. A similar case was also recorded at a City of Harare Council farm (Harare) in which ten cases of anthrax in cattle were spread over a month between October and November 2015 [44]. Further, changes in the seasonality of anthrax outbreaks have been observed in Kruger National Park, South Africa, in which incidents of the disease were noted during the rainy season (December–March) [45]. Incidents of anthrax outbreaks during the rainy season were similarly noted in Zimbabwe. These variations in anthrax patterns call for vigilance at all times and for veterinarians and human health personnel to recognize the disease should it appear ‘out of season’ and out of character.

According to the Department of Epidemiology and Disease Control, Ministry of Health & Child Care (Zimbabwe) all cases of cutaneous and enteric anthrax noted in humans in this study were due to contracting of the disease from livestock, including missed cases in livestock. Given that about 10.5% of the disease outbreaks in livestock also involved at least a human case, and the number of human cases of anthrax between 2010 and 2018 noted in this study, this would add further evidence to a significant under reporting of the disease in animals in consonant with the global trends [13,46].

Notwithstanding sub-optimal disease surveillance and reporting, our data indicated that anthrax caused a significant high mortality in both livestock (particularly cattle) and wildlife between 2000 and 2018. In agreement with another study at interface sites in Zimbabwe [47], communities at the study sites also indicated that anthrax was an economically important livestock disease alongside others such as lumpy skin disease, black leg and foot and mouth disease. Hence, the disease may have an economic impact on the national livestock herd, but which is felt more acutely at an individual household level in communal lands where the family’s wealth is held in cattle. This is exemplified by two households encountered during the survey in Chizvirizvi site who respectively lost 75% (15/20) and 100% (11/11) of their cattle during the 2004–2005 anthrax outbreaks. Another household in Magunje lost all 9 (100%) of its cattle due to two anthrax outbreaks between 2008 and 2012. Hence, the contribution of the disease to poverty was apparent [48] but felt more acutely at a household level. Similarly, the increasing impact of anthrax on wildlife populations may ultimately affect species conservation efforts with loss of goods and services for ecotourism. Thus surveillance and preventive measures for the disease need prioritizing in order to prevent these negative impacts.

Some potential biases of the study approaches need to be considered. Firstly, the questionnaire was not designed in the respective vernacular languages of the respondents and hence, language bias could not be ruled out. However, depending on the interviewee’s vernacular language preference, the interpretation was aided by the local animal health extension officers who had lived in the study sites for years. Secondly, respondents were requested to give a detailed account of anthrax outbreaks in the last ten years before 2015 and a recall bias could be one of the study limitations. Nevertheless, triangulation was employed to reduce the negative impact of this bias on our study results. Thirdly, the findings of this study were limited by the type of disease data used, which were collected passively. Such data make it hard to note missing pieces of data due to underreporting or failure by staff to electronically capture the data. Although anthrax is a reportable disease in Zimbabwe, not all outbreaks are reported; hence there is a possibility of some bias in the collected data. Furthermore, as indicated by interviewees in this study, some livestock anthrax oubreaks were detected as traceback from human anthrax cases. Therefore, anthrax outbreaks detailed in this study include only those that were reported and only provide an index of the magnitude of the disease which is probably an underestimate of the extent of the problem. Nevertheless, despite these limitations, the study results highlight research gaps and provide information that can be used to develop appropriate surveillance and control strategies for anthrax in the country.

In conclusion, we demonstratethat anthrax outbreak patterns in Zimbabwe are on the rise and the disease is spreading throughout the country with some areas previously designated as low anthrax zones now experiencing more outbreaks. This was more revealing in wildlife anthrax outbreaks particularly along the Zambezi River basin. Control, management and surveillance efforts on the disease were noted to be inadequate. The results imply that there is need to review the anthrax vaccination strategies and anthrax areas designated as high, medium and low risk zones. Improvement on institutional collaboration and linkages of the veterinary, wildlife and human medical services to enable holistic and coordinated disease surveillance, monitoring, management, control and preventive measures under the One Health Concept is recommended. The results showed that there is need to educate veterinary and human medical extension staff, rural livestock farmers and wildlife management personnel on the changing patterns of the disease, its recognition and early reporting. While the national legislation was in place for control of anthrax including carass disposal and decontamination of the environment following outbreaks of the disease, the enforcement of this legislation needs improvement. Despite the shortcomings of the surveillance system employed, the present paper illustrates the benefits of collecting long-term livestock, wildlife and human health data and such a system, with modifications can be implemented in other developing countries. The analysis employed here to depict anthrax outbreak patterns is simple and may be replicated in other countries to investigate changes in disease patterns with respect to space and time.
